# Avoidable injuries in pediatric hand fractures: a 30-year review of compensation claims in Finland

**DOI:** 10.2340/17453674.2025.43084

**Published:** 2025-03-31

**Authors:** Caroline C DIKERT, Petra GRAHN, Yrjänä NIETOSVAARA

**Affiliations:** 1 Department of Pediatric Orthopedics and Traumatology, New Children’s Hospital, Helsinki University Hospital, University of Helsinki, Helsinki; 2 Department of Pediatric Surgery, Kuopio University Hospital, University of Eastern Finland, Kuopio, Finland

## Abstract

**Background and purpose:**

We aimed to evaluate the risk, causes, and compensation outcomes of avoidable injuries in pediatric hand fractures.

**Methods:**

All compensation claims submitted to the Finnish Patient Insurance Centre (PIC) for pediatric hand fracture treatment (< 16 years) in Finland from 1990 to 2019 were reviewed. Data collected included fracture location, compensation rate and reasons, treatment setting, and treating professional specialty. PIC decisions were reassessed. Census population (3.0 x 10^7^) was calculated from national registers, and hand fracture incidence estimated (350/10^5^).

**Results:**

Of 101 claims, 71 were compensated as avoidable injuries, encompassing 72 complications by 74 professionals. Compensated claims were most common for finger fractures (43/65), followed by metacarpal (15/20) and scaphoid fractures (14/17). 1 claim involved both a finger and metacarpal fracture, and in 3 claims faults were made by 2 separate professionals. Most injuries occurred in healthcare centers, with general practitioners responsible for 37/74 avoidable injuries. Diagnostic delays led to most compensation (36 cases: 23 lacked initial radiographs, 12 missed fractures on radiographs). Compensation for permanent disability (5–10%) was granted in 8 cases (4 finger and 4 scaphoid fractures) and for cosmetic disability in 21/43 finger fracture cases. PIC decisions were deemed correct in 98/101 cases, with a calculated risk of compensated avoidable injury at 0.1%.

**Conclusion:**

The risk of avoidable injuries with permanent sequelae in pediatric hand fractures in Finland is low. Use of diagnostic radiographs is advised in children with hand injuries, especially if scaphoid fractures are suspected.

Fractures of the hand are the second most common fracture location in the pediatric population with an estimated incidence of 350 per 100,000 [1-3]. Previous studies suggest that 59–78% of hand fractures are finger fractures and 40% of fractures occur in the fifth ray [1,2,4-6]. Most pediatric hand fractures can be treated nonoperatively [4,6,7].

The Finnish Patient Insurance Centre (PIC) handles all compensation claims related to potential avoidable injuries following medical treatment in Finland. Claims must be submitted to PIC within 3 years from when the patient became, or should have become, aware of the injury [8]. If the claimant is dissatisfied with either PIC’s decision or the amount of compensation, they may request a re-evaluation by the Traffic Accident and Patient Injuries Board within 1 year of PIC’s decision [8]. Permanent disability is assessed in 5% increments, categorized into classes 1–20, based on guidelines provided by the Traffic and Patient Accident Board [9]. The risk of avoidable injuries, including temporary discomfort, permanent functional, and/or cosmetic disability, in the management of pediatric hand fractures has not been reported before. The purpose of this study is to evaluate risk, causes, and compensation outcomes of avoidable injuries in pediatric hand fractures in Finland, with a focus on diagnostic delays and the use of radiographs.

## Methods

### Design

This is a retrospective study on compensations claims related to management of hand fractures during 1990–2019 in children less than 16 years old in Finland. An independent observer (pediatric hand surgeon) and a PIC medical adviser (pediatric hand surgeon) re-evaluated the written claims retrospectively to assess whether the decisions of PIC were correct.

The study is reported according to STROBE guidelines [10].

### Population

Patients were identified using International Statistical Classification of Diseases and Related Health Problems (ICD) codes [11]. Both ICD-9 and ICD-10 diagnosis and procedure codes were used. The utilized diagnosis codes for wrist and hand fractures were 814–817 and S62. The procedural codes (procedures on the bones of the wrist and hand, and removal of implants and external fixation devices) were 912, 913, 916 and NDJ, NDK, NDU. All subgroups of the mentioned diagnosis and procedural codes were included. The mean annual census population in Finland during the study period was 985,029 (range 931,508–1,036,791; SD 35,045) [12].

### Outcomes

Information collected from the treatment claims included patient age, date of injury, place and method of treatment, and the specialty of the treating medical professional. Data was also gathered on the reasons for claims, grounds for compensation, and any client complaints regarding PIC’s decision to the Traffic and Patient Accident Board. The incidence of an avoidable injury was calculated based on national registers.

### Statistics

The difference between means (MD) was calculated with a 2-sample t-test and a 95% confidence interval (CI) was used to express differences between continuous data.

### Ethics, use of AI tools, funding, and disclosures

Approval for the study was obtained from the Finnish Patient Insurance Centre (www.pvk.fi). None of the authors received any funding for the study. No AI tools were used. YN reports a conflict of interest as he serves as a medical adviser for the PIC. Complete disclosure of interest forms according to ICMJE are available on the article page, doi: 10.2340/17453674.2025.43084

## Results

**Figure 1:**
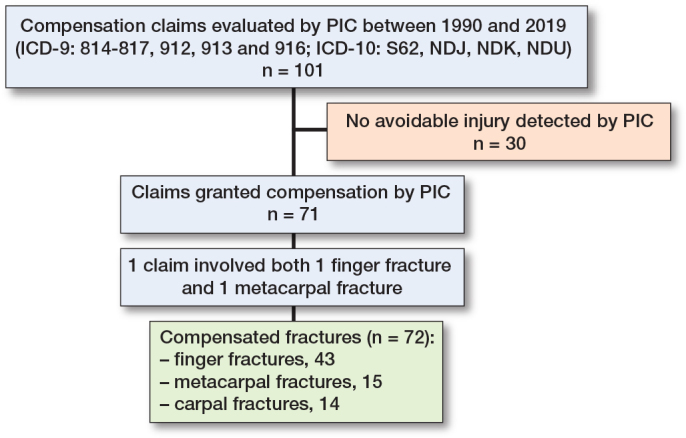
Flowchart of cases included in the study. PIC = Patient Insurance Centre, ICD = International Statistical Classification of Diseases and Related Health Problems.

**Figure 2:**
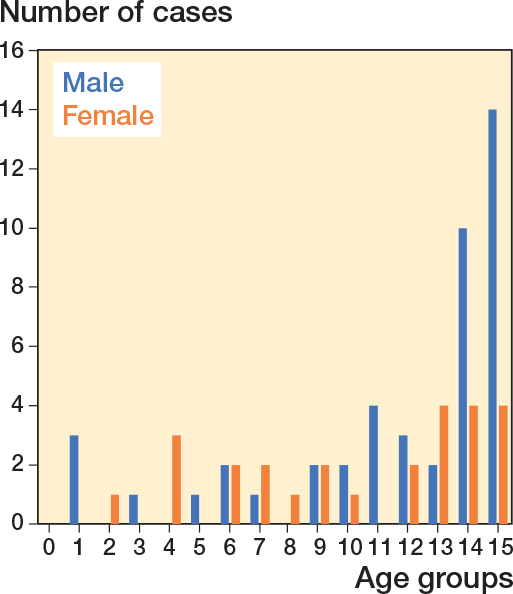
Age and sex of the 71 patients, at the time of their fracture, who received compensation for an avoidable injury following treatment of hand fractures in Finland during 1990–2019.

Of the 101 claims submitted over the study period, 71 applicants were granted compensation for a total of 72 avoidable injuries (1 claim involved 2 different avoidable injuries), caused by 74 different professionals (2 fractures missed twice) (Figure 1). Radiographs were available for the adviser at PIC in 93 cases and photographs in 26 cases. The mean age of the patients with an avoidable injury was 11 years (range 1–15, SD 4.3), and the injuries were most frequent at age 15 (Figure 2). Of the 71 cases, 36 involved the left extremity and 44 occurred in male patients. Male patients were older at the time of injury (mean 11.6 years, SD 4.2 vs female mean 10.3 years, SD 4.1, MD CI –0.7 to 3.4) (Figure 2). 37 of the 74 faults made by medical professionals occurred in healthcare centers, 14 in central hospitals, 10 in university hospitals, 9 in city or district hospitals, and 4 in private clinics. 37 avoidable injuries were caused by general practitioners (Table 1). Diagnostic delay (36 cases), primarily due to absence of a primary radiograph (23 cases), or failure to detect the fracture in a radiograph (12 cases) were the most common reasons for granted compensation.

**Table 1:** Factors contributing to Patient Insurance Centre (PIC) compensated treatment claims in 71 children less than 16 years of age in Finland between 1990 and 2019

Specialty	Diagnostic delay	Inappropriate closed treatment	Unsatisfactory standard of surgery	All
General practitioner	19	18	0	37
Surgical trainee	7	4	2	13
Orthopedic surgeon	2	1	2	5
Radiologist	4	0	0	4
Pediatric surgeon	0	0	2	2
General surgeon	0	1	0	1
Hand surgeon	0	1	0	1
Nurse	3	0	0	3
Medical student	1	0	0	1
Not known	2	3	2	7
All professions	38	28	8	74

2 clients received compensation for 2 different episodes of avoidable injuries leading to diagnostic delay, and 1 for both diagnostic delay and unsatisfactory standard of surgery.

### Finger fractures

**Figure 3:**
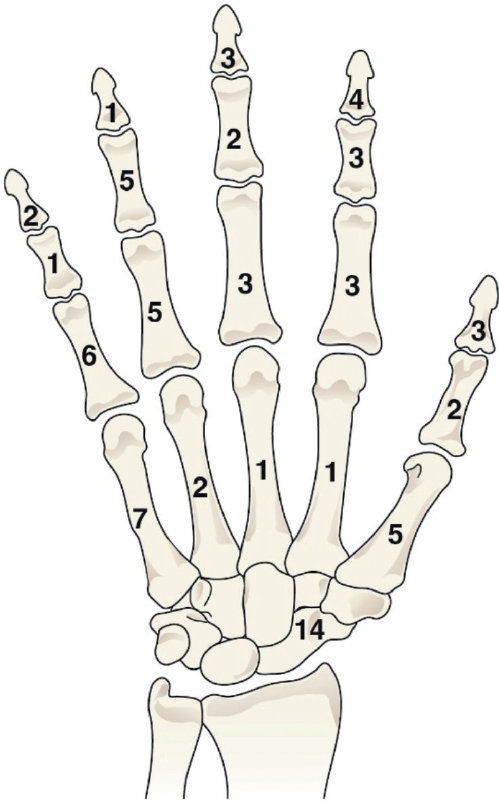
Distribution of the 73 fractures in the 71 children who received monetary compensation from the Finnish Patient Insurance Centre for an avoidable injury of their hand fracture in Finland during 1990–2019.

Compensation was granted for 45 complications occurring in 43 finger fractures, due to a double fault in 2 injuries (Figure 3, Table 2). The mean age of the patients with a compensated finger fracture was 9.3 years (range 1–15, SD 4.4). Both sexes and sides were equally affected (male 22/43, left 23/43) with no noteworthy difference between the rays (Figure 2). Of the 45 avoidable injuries, 23 occurred in health care centers, 9 in central hospitals, 5 in city or district hospitals, 4 in private clinics, and another 4 in university hospitals. Half (23/45) of the injuries were caused by general practitioners. Compensation was granted for inappropriate closed treatment in 20 of the 43 fractures, for diagnostic delays in 19 (including 13 without an initial radiograph, 5 with missed fractures on radiographs, and 1 due to another reason), and for inadequate surgical standards in 4. In 2 of the 43 cases the diagnostic delay was caused by both a general practitioner and a radiologist.

**Table 2:** Patient Insurance Centre claims and compensated avoidable injuries in relation to fracture location in 101 children less than 16 years of age in Finland between 1990 and 2019

Fracture location	Claims	Compensated	Diagnostic delay	Inappropriate closed treatment	Unsatisfactory standard of surgery	Permanent functional disability	Permanent cosmetic disability
Finger	65	43	19	20	4	4	21
Metacarpal	20	15	4	8	3	0	4
Scaphoid	17	14	14	0	1	4	7
All	101^a^	71^a^	36^a^	28	8	8	32

a 1 case involved both a finger and a metacarpal fracture.

PIC determined a 5% permanent functional disability in 4 fractures, which was attributed to reduced range of motion in all 4 cases and permanent deformity in 2 (Table 2). Permanent cosmetic disability was compensated in 21 of the 43 cases.

### Metacarpal fractures

Avoidable injuries were identified in 16 metacarpal fractures (Figure 3, Table 2). 1 claim included 2 metacarpal fractures, resulting in a total of 15 compensations. The mean age of patients with a compensated metacarpal fracture was 13.6 years (range 5–15, SD 2.4). Of the 15 avoidable injuries, 13 were in males, and 11 involved the right hand. The fifth metacarpal was most often affected (Figure 2). Avoidable injuries were most frequently reported in healthcare centers, with general practitioners responsible in 6 cases. Of the remaining cases, 5 occurred in university hospitals, 2 in district hospitals, and 2 in central hospitals. Compensation was granted due to inappropriate closed treatment in 8 cases, diagnostic delay in 4 cases (3 with no initial radiograph, 1 with a missed fracture on radiograph), and inadequate surgical standards in 3. None of these children received compensation for permanent functional disability, while 4 were compensated for permanent cosmetic disability (Table 2).

### Carpal fractures

14 children with scaphoid fractures were compensated for a total of 15 avoidable injuries (Figure 3, Table 2). The mean age of patients with avoidable injuries was 14.1 years (range 11–15, SD 1.4). 10 patients were male, and 8 cases involved the left hand. 9 of the injuries occurred in healthcare centers, 3 in central hospitals, 2 in city or district hospitals, and 1 in a university hospital. General practitioners were found to be at fault in 9 cases. None of the scaphoid fractures were diagnosed during the initial evaluation, with 9 missed in primary radiographs and 5 cases lacking initial radiographs altogether. All scaphoid fractures received compensation due to delayed diagnosis, and 1 case additionally for inadequate surgical standards. The PIC identified permanent functional disability in 4 cases following an avoidable injury: 3 with a 10% disability and 1 with a 5% disability, all due to reduced range of motion. Additionally, 2 patients reported ongoing pain, and 1 experienced reduced grip strength. 7 patients received compensation for permanent cosmetic disability (Table 2).

### Incidence of avoidable injuries

**Figure 4:**
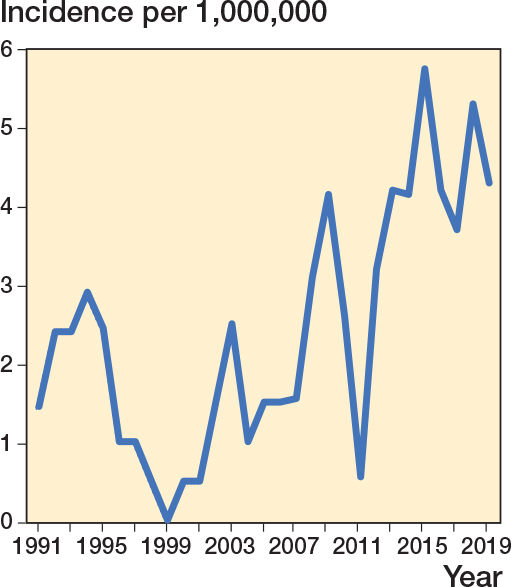
Calculated annual incidence of compensated avoidable injuries following management of hand fractures per 10^6^ in children less than 16 years of age in Finland expressed as a 2-point moving average.

The calculated incidence for an avoidable injury for pediatric hand fractures was 2.4 per 1,000,000 (range 0–6.3, SD 1.9) and increased over time (Figure 4). The mean annual incidence for an avoidable injury was 1.5 per 1,000,000 (range 0–4.3, SD 1.4) after a finger fracture, 0.5 per 1,000,000 (range 0–3.1, SD 0.8) after a metacarpal fracture, and 0.5 per 1,000,000 (range 0–2.1, SD 0.7) after a scaphoid fracture.

### PIC decision and expert opinion

The PIC medical adviser evaluating the claims was a pediatric orthopedic surgeon in 45 cases, an orthopedic surgeon in 20, a hand surgeon in 18, a general practitioner in 14, a general surgeon in 2, a specialist in public health in 1, and of unknown profession in 1 case. There were no complaints concerning the decisions made by the medical adviser at PIC, and therefore no cases were evaluated by the Traffic Accident and Patient Injuries Board. On retrospective evaluation both a medical adviser working for PIC (YN) and an independent expert (PG) agreed with PIC’s decision regarding compensation in 98 of the 101 cases. In 1 case they would have granted compensation for a different reason and in 4 they found that compensation should have been granted for only a 5% functional handicap instead of 10% as granted by the medical adviser. 3 treatment injuries involved unnecessary amputation of the distal phalanx, which the experts considered a significant harm.

## Discussion

We evaluated pediatric hand injuries and showed that the risk of an avoidable injury following a pediatric hand fracture is low, with 36 of 71 cases in the study potentially preventable.

In previous studies evaluating avoidable injuries, 71% of claims regarding complications after a pediatric distal humeral fracture and 64% of claims regarding a pediatric tibial fracture were granted compensation by PIC [13-15]. This is consistent with the rate we discovered among hand fractures (70%). Additionally, the distribution of fractures in the complaints we examined aligns with the reported pattern of pediatric hand fractures [4,16]. Male patients account for 62–75% of pediatric hand fractures and tend to be older at the time of injury (male mean age 11.3 years, SD 3.5; female mean age 10.6 years, SD 3.5) [2-4,6]. This is consistent with our study, where males were older (mean age 11.6 years) and represented 62% of the cohort. Pediatric hand fractures appear to affect both sides equally (right hand 55.1%, right-to-left ratio 1.2), which also applied to our findings [2,4]. However, complications were more evenly distributed across different fracture locations compared with the general distribution of pediatric hand fractures, where the fifth ray is injured in 40% of cases (20% avoidable injuries) [2,4].

A study at Boston Children’s Hospital in the USA found that 57% of patients initially presented to an outside provider or institution, and they saw 1–2 providers before consulting a hand surgeon [6]. The referral pattern for pediatric hand fractures in Finland is similar, with patients typically seeing a similar number of providers before reaching a surgical specialist. Patients may initially receive treatment in primary care or the private sector before being referred to the emergency department or directly to a surgical specialist, either urgently or non-urgently. Surgical treatment is required for unstable or non-reducible fractures, intra-articular fractures, and open fractures in approximately 10–15% of finger fractures [4,6]. In 12 out of 20 finger fractures compensated for inappropriate closed treatment, the need for either closed reduction or surgical treatment was unrecognized. We recommend increasing primary care knowledge on when finger fractures should be managed with closed reduction and when surgical intervention is warranted.

In our study, scaphoid fractures accounted for nearly one-fourth of the compensated cases (15/71), with all cases compensated due to diagnostic delays. Among adults, 92% of avoidable injuries from scaphoid fractures are attributed to delayed diagnosis, with 60% leading to permanent functional disability [17]. In contrast, the risk of permanent functional disability following an avoidable injury was lower in the pediatric population (29%). Additionally, scaphoid fractures represented a higher proportion of all avoidable injuries (20%) compared with their general incidence among hand fractures (0.3–6%) and had a threefold risk (29% vs 11%) of resulting in permanent functional disability compared with other pediatric hand fractures [1,2,4]. The main reason (51%, 36/71) for granted compensations in pediatric hand fractures was a delay in diagnosis, primarily due to a lack of primary radiographs (23/36). In a study analyzing avoidable injuries after pediatric tibial fractures, 20% were due to the absence of a primary radiograph [14]. The threshold for taking radiographs in children presenting with trauma appears to be too high, and many injuries could likely be prevented by obtaining basic radiographs. The radiation dose for an extremity radiograph is 0.01 mSv [18], which is very low compared with the annual environmental radiation in Finland, which is 5 mSv [19].

The estimated annual incidence of pediatric hand fractures is 350 per 100,000, corresponding to a 0.07% risk of an avoidable injury following treatment [1-3,16]. This risk is notably lower than for other pediatric fractures: 0.4% after a pediatric tibial fracture, 1.1% after a pediatric distal humeral fracture, and 2.2% after a pediatric femoral fracture [13-15]. This difference likely stems from the fact that most pediatric hand fractures heal without complications and seldom require surgical intervention [4,6]. Furthermore, pediatric hand fractures rarely involve growth-plate injuries that would result in growth arrest, while distal humeral, tibial, and femoral fractures often affect the growth plate, leading to potential length discrepancies, angulation, or limited range of motion.

The number of patient injury claims has increased over recent decades, a trend reflected in our study [8]. This rise may be due in part to the growing number of procedures performed, along with increased public awareness of patient insurance. Although the typical proportion of compensated claims since 1990 has been around one-third to one-fourth, in this study, 71 out of 101 claims were compensated, suggesting that less severe cases are underrepresented [8].

The actual number of avoidable injuries may be higher than reported, as not all injuries are evaluated by PIC—some patients may lack information or be unwilling to file a complaint. These issues and the retrospective nature of the study account for the limitations of the study. The study is strengthened by its nationwide scope, spanning 3 decades, which provides a comprehensive view of these trends in Finland.

### Conclusions

Avoidable injuries following pediatric hand fractures are rare, with a low risk of permanent functional disability. Improving the diagnostics of hand fractures, particularly scaphoid fractures, and increasing primary care knowledge of surgical indications for pediatric finger fractures could help reduce these injuries.

## Notes

YN designed the study, served as a medical expert and revised the paper. CD collected and analysed the data and wrote the paper. PG served as a medical expert and contributed to writing and revising the paper.

Handling co-editors: Ivan Hvid and Robin Christensen

*Acta* thanks Pelle Gustafson and Georg Singer for help with peer review of this manuscript.
